# Is Statistical Learning of a Salient Distractor’s Color Implicit, Inflexible and Distinct From Inter-Trial Priming?

**DOI:** 10.5334/joc.243

**Published:** 2022-10-17

**Authors:** Aidai Golan, Dominique Lamy

**Affiliations:** 1 School of Psychological Sciences, Tel Aviv University, IL; 2 Sagol School of Neuroscience, Tel Aviv University, IL

**Keywords:** Color suppression, statistical learning, attentional guidance, awareness, intertrial priming, selection history, visual search

## Abstract

Being able to overcome distraction by salient distractors is critical in order to allocate our attention efficiently. Previous research showed that observers can learn to ignore salient distractors endowed with some regularity, such as a high-probability location or feature – a phenomenon known as distractor statistical learning. Unlike goal-directed attentional guidance, the bias induced by statistical learning is thought to be implicit, long-lasting and inflexible. We tested these claims with regard to statistical learning of distractor color in a high-power (N = 160) pre-registered experiment. Participants searched for a known-shape singleton target and a color singleton distractor, when present, appeared most often in one color during the learning phase, but equally often in all possible colors during the extinction phase. We used a sensitive measure of participants’ awareness of the probability manipulation. The awareness test was administered after the extinction phase for one group, and after the leaning phase for another group – which was informed that the probability imbalance would be discontinued in the upcoming extinction phase. Participants learned to suppress the high-probability distractor color very fast, an effect partly due to intertrial priming. Crucially, there was only little evidence that the bias survived during extinction. Awareness of the manipulation was associated with *reduced* color suppression, suggesting that the bias was implicit. Finally, results showed that the awareness test was more sensitive when administered early vs. late. We conclude that learnt color suppression is an implicit bias that emerges and decays rapidly, and discuss the methodological implications of our findings.

## Introduction

Our ability to ignore salient but irrelevant information has become especially important in the Internet age, as many of our everyday offline tasks have moved online. Consider shopping or reading the news on our cellphone. In order to complete these tasks effectively, we need to focus on the items of potential interest and ignore salient irrelevant items, such as flashing advertisement banners or promotional videos.

How can salient distractors be ignored^1^? There is no consensus as to whether telling an observer where a salient distractor will appear and how it will look like, is useful (e.g., Arita, Carlisle & Woodman, 2012; Carlisle & Woodman, 2011; Chang & Egeth, 2019; Chao, 2010; Conci et al., 2019; Geng, 2014; Geng, Won & Carlisle, 2019; Heuer & Schubo, 2020; Munneke, der Stigchel & Theeuwes, 2008; Munneke et al., 2011; Ruff & Driver, 2006; Zhang, Gaspelin & Carlisle, 2020) or ineffective (e.g., Becker, Hemsteger & Peltier, 2015; Berggren & Eimer, 2021, Chellazzi et al., 2019; Cunningham & Egeth, 2016; Moher & Egeth, 2012; Noonan et al., 2016, 2018; Wang & Theeuwes, 2018a). By contrast, it is widely agreed that one can learn to ignore a salient distractor through experience, without being explicitly informed of its most probable location or feature, a phenomenon known as statistical learning.

**Figure 1:**
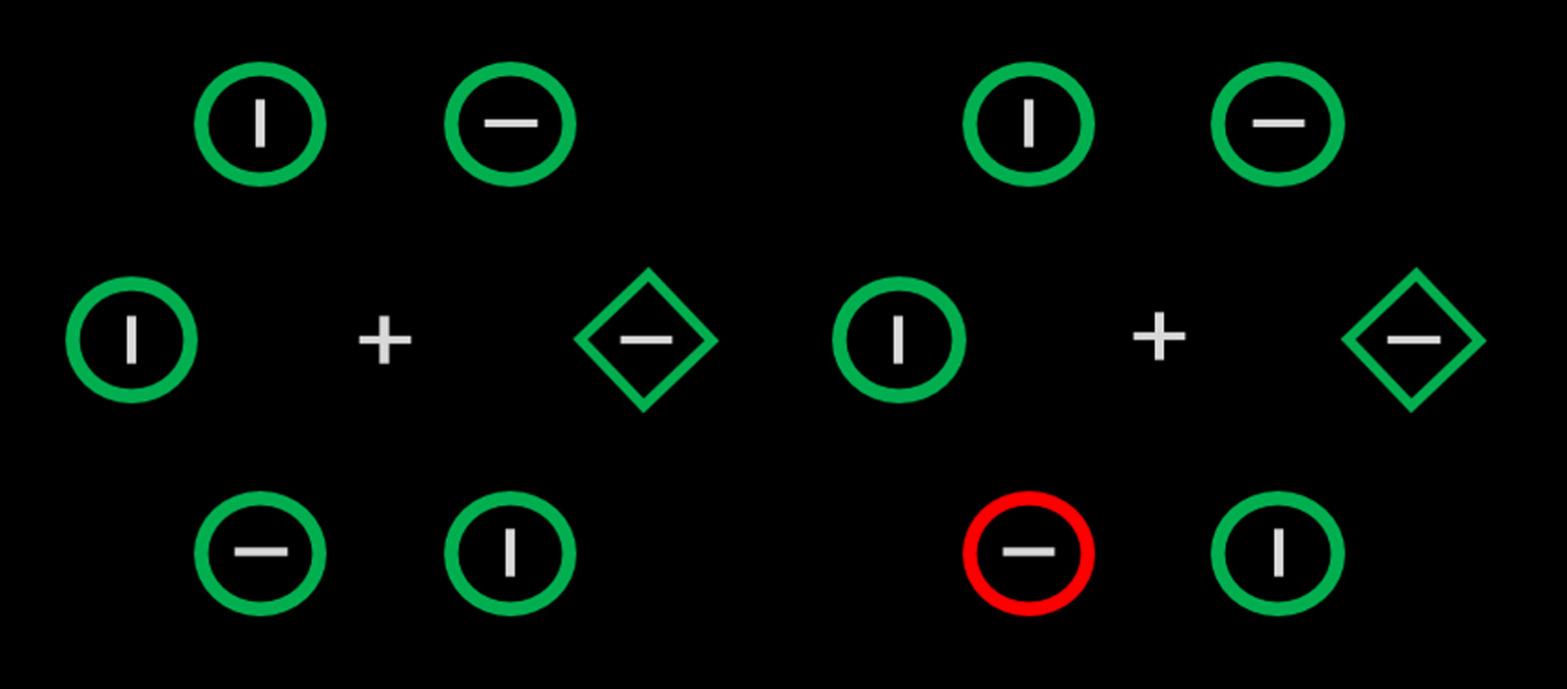
Sample displays in a typical additional singleton task. Participants search for the diamond, and the distractor (the unique red object) is either absent (left) or present (right).

Statistical learning studies that investigated learned distractor suppression most often relied on the additional singleton paradigm (see Figure 1). In this paradigm, the search target is the odd shape (e.g., a diamond among circles) and one of the distractors has a unique color (e.g., green among red). The critical probability manipulation consists in having the color-singleton distractor appear more often in one location than in other locations or in one color than in the others (high- vs. low-probability locations or colors, respectively). The typical finding is that the salient distractor interferes less with performance when it appears at the high- vs. at a low-probability location (e.g., Britton & Anderson, 2020; Di Caro, Theeuwes & Libera, 2019; Duncan & Theeuwes, 2020; Failing et al., 2019; Failing, Wang & Theeuwes, 2019; Failing & Theeuwes, 2020; Ferrante et al., 2018; Huang et al., 2021; Gong & Theeuwes, 2021; Goschy et al., 2014; Kong et al., 2020; Moorselaar et al., 2020; Wang & Theeuwes, 2018a; 2018b; 2018c; Wang et al., 2019; Sauter et al., 2018; 2019; 2021; Zhang, Gapelin & Carlisle, 2019) and when it takes on the high- vs. a low-probability color (e.g., Failing et al., 2019; Stilwell, Bahle & Vecera, 2019, but see Wang & Theeuwes, 2018c). The conclusion from these findings is that participants learn to suppress the locations or features that reliably characterize potentially distracting objects.

Learning in these studies is thought to be implicit and inflexible, two features that distinguish attentional guidance by statistical learning from top-down attentional guidance (e.g., Awh, Belopolsky & Theeuwes, 2012; Theeuwes, 2018; Jiang, 2018). When explicitly probed at the end of the experiment, participants typically fail to correctly indicate the high-probability location or feature (e.g., Ferrante et al., 2018; Failing et al., 2019); this finding is taken to indicate that participants have no conscious awareness of the learnt statistical regularity that influenced their behavior. In addition, when the probability imbalance that prevailed in the learning phase is discontinued in an extinction phase that immediately follows, participants remain biased towards the high-probability location (Britton & Anderson, 2020; Duncan & Theeuwes, 2020; Valsecchi & Turatto, 2021); the fact that the learnt bias persists even when it is no longer useful is taken to indicate that participants have no control over it.

Finally, statistical learning is also thought to be distinct from intertrial priming of distractor suppression. Intertrial priming refers to the finding that it is easier to ignore a distractor when its location or feature repeats from the previous trial than when it changes (e.g., Goschy et al., 2014 and Meeter & Olivers, 2006, Exp.3, respectively). When the distractor is more likely to appear at one location or to have a given feature, this location or feature is necessarily also more likely to repeat than to change on consecutive trials. To demonstrate that statistical learning operates over longer time frames and cannot be entirely accounted for by inter-trial priming, some researchers remove the trials on which a repetition occurred from the previous trial (e.g., Failing & Theeuwes, 2019; Failing et al., 2019 for distractor-location statistical learning; Stilwell et al., 2019 for distractor-feature statistical learning; but see Moorselaar, Daneshtalab & Slagter, 2021). Others rely on the data from the extinction phase, in which the distractor location or feature is no longer more likely than chance to repeat on consecutive trials (e.g., Britton & Anderson, 2020; Duncan & Theeuwes, 2020; Ferrante et al., 2018; Valsecchi & Turatto, 2021).

### Is learned distractor suppression implicit, inflexible and distinct from inter-trial priming?

There are several reasons to reevaluate the conclusion that statistical learning of distractor suppression is implicit, inflexible and distinct from inter-trial priming. First, consider how awareness of the statistical manipulation is usually tested. At the end of the experiment, participants are asked whether they noticed any regularity during the learning phase; then, they are required to make just one forced-choice decision: for instance, for statistical learning of distractor location, they are asked to guess where the distractor had most often appeared. If fewer participants than could be expected by chance select the correct answer, the conclusion is that participants were unaware of the statistical manipulation. In most cases (e.g., Failing & Theeuwes, 2019; Ferrante et al., 2018; Moorselaar et al., 2021), the few participants who guessed right are removed from the analysis.

Relying on such awareness tests to infer that learning is implicit has been heavily criticized in the context of related implicit-learning phenomena (e.g., contextual cueing, Vadillo, Konstantinidis & Shanks, 2016; target location probability learning, Vadillo et al., 2020; Gimenez-Fernandez et al., 2020). Vadillo and colleagues suggested that the single-trial forced-choice procedure is not sensitive enough to detect partial awareness of the probability manipulation. In line with this conjecture, they showed that awareness is correlated with target-location statistical learning when more sensitive tests are used to probe awareness, namely, when participants are asked to estimate on what percentage of the trials the target had appeared in each location^2^ (Gimenez-Fernandez et al., 2020, Exp.2). In addition, these authors criticized the timing of the awareness test: assessing awareness after the extinction phase is likely to dilute the manipulation’s impact on the forced-choice report, because the test comes following many trials in which the statistical manipulation was no longer present. Corroborating this claim, Gimenez-Fernandez et al. (2020) reported that awareness of the statistical manipulation was significantly higher when the test was administered immediately after the learning phase than after the extinction phase (d = .50). Finally, Shanks (2017) warned against excluding participants who are aware of the concealed information, because of regression-to-the-mean issues.

Second, consider how the two-phase, learning-extinction, design is implemented in order to demonstrate that statistical learning is inflexible. The statistical imbalance present in the learning phase is discontinued in the extinction phase, but participants are not informed of this transition in any way (Britton & Anderson, 2020; Duncan & Theeuwes, 2020; Valsecchi & Turatto, 2021). This is a weak test of flexibility, because participants are not given the opportunity to purposefully stop relying on the learned statistical bias. A stronger test of flexibility would be to inform participants before the extinction phase that there was a probability imbalance during the learning phase and that it will be discontinued in the extinction phase that follows. In other words, to demonstrate that statistical learning of distractor suppression is inflexible, one would have to show that the bias against the distractor’s previously probable location or feature is still observed in the extinction phase, despite participants being explicitly informed that the statistical manipulation will be absent.

Finally, because the influence of inter-trial repetitions typically lasts over 5–8 trials (Maljkovic & Nakayama, 1994; 1996), removing only repetitions from the previous trial is not enough to invalidate intertrial priming as an alternative account to statistical learning. It is preferable to use the learning-extinction design for this purpose, because any performance advantage in the extinction phase for the location or feature that was highly probable during learning cannot be attributed to short-term inter-trial priming.

### Learned suppression of high-probability distractor features

Many studies showed that distractor-feature regularities are learned during visual search (e.g., Gaspelin, Leonard & Luck, 2015; Gaspelin & Luck, 2018a, 2018b; Graves & Egeth, 2015; Vatterott, Mozer & Vecera, 2018; Vatterott & Vecera, 2012). However, only few studies took steps to investigate whether such learned distractor-feature suppression can be distinguished from top-down suppression or inter-trial priming (Failing et al., 2019; Moorselaar et al., 2020; Moorselaar et al., 2021; Stilwell, Bahle & Vecera, 2019; Wang & Theeuwes, 2018c). Only one study probed participants’ awareness (Failing et al., 2019), but it used a variant of the single-trial forced choice procedure: only participants who correctly indicated the locations where two different distractors had appeared with higher probability were considered to be “aware”. Two studies controlled for inter-trial priming by removing repeated-feature trials (Stilwell et al., 2019; Failing et al., 2019), and none used an extinction phase.

### The present study

The objective of the present study was to determine the extent to which attentional guidance by statistical learning of a salient distractor’s feature is implicit, inflexible and distinct from intertrial priming. This study was pre-registered. Similar to Stilwell et al. (2019, Exp.2), we used the additional singleton paradigm and manipulated the probability of the singleton distractor’s color. On each trial of the learning phase, the color singleton took on one of five possible colors, with one color (high-probability color) being more likely than the others (low-probability colors).

The learning phase was followed by an extinction phase, during which all singleton colors appeared equiprobably. At the end of the extinction phase, to test participants’ awareness of the probability manipulation, we asked them to estimate the proportion of trials in which the distractor had appeared in each color – a procedure that was shown to be the most sensitive in statistical learning tasks (Gimenez-Fernandez et al., 2020).

Crucially, while one group of participants took the awareness test immediately after the extinction phase, as is typical of most previous statistical learning studies (e.g., Jiang et al., 2013, Ferrante et al., 2018), the other group took the test immediately after the learning phase. In that group, participants were also told about the distractor-color probability manipulation implemented during the learning phase and were explicitly informed that this manipulation would be discontinued during the upcoming (extinction) phase. In the remainder of this paper, we refer to these two groups as the *early*- and *late-awareness-test* group for results related to the awareness test, and as the *informed* and *uninformed* group, respectively, for results related to participants’ voluntary control over the bias.

### Pre-registered predictions

We had three main predictions with regard to the learning phase. First, we expected the interference from the color singleton distractor (i.e., the performance cost when the singleton was present vs. absent) to be smaller when this distractor appeared in the high- than in a low-probability color, as was observed in previous studies (e.g., Stilwell et al., 2019; Failing et al., 2019). In the remainder of this paper, we refer to the difference in performance between low- and high-probability color singleton trials as the *color-suppression effect*. Second, we expected color-suppression to develop across the learning phase – a question that was not addressed in previous studies. Finally, as the learning phase was identical for informed and for uninformed participants, we expected no difference between these groups during learning.

For the extinction phase, we assessed the benefit of repeating the color of the singleton distractor from the previous trial as well as from trials further back: since in the learning phase, such intertrial priming, if present, was more likely to occur for the high- than for the low-probability colors, this analysis allowed us to evaluate the contribution of intertrial priming in the color-suppression effect observed during the learning phase.^3^ Crucially, we examined whether a color suppression effect would still be observed during the extinction phase: finding that it is would indicate that color-suppression reflects (long-term) statistical learning rather than only (short-term) inter-trial priming.

To assess the extent to which learned suppression of the distractor’s high-probability color is flexible, we compared the magnitude and duration of the color-suppression effect during the extinction phase between the informed and uninformed groups. If participants have some control over the learnt bias, we expected the color-suppression effect to be smaller and wane faster across the extinction phase in the informed than in the uninformed group. Conversely, we expected no difference between the groups if statistical learning of the distractor’s color is not flexible.

Finally, if statistical learning depends on conscious awareness of the probability manipulation, then the color-suppression effect during learning should be larger in participants with higher probability estimates for the high-probability color. Conversely, if learning is implicit, we expected no correlation between the two measures.

Our last predictions concerned the sensitivity of the awareness measure taken before vs. after the extinction phase. If testing awareness after the extinction phase indeed provides a less sensitive measure as suggested by Gimenez-Fernandez et al. (2020), participants should estimate that the high-probability color occurred more often in the early- than in the late-awareness-test group. In addition, the correlation between these probability ratings and the color-suppression effect, if any, should be weaker in the latter than in the former group.

## Methods

### Sample size selection

Because Stilwell et al. (2019) did not report the effect size of the color-suppression effect, we used the distractor-location probability effect size reported in the online study by Duncan and Theeuwes (2020, Exp. 3) in order to determine the number of participants required in our study. The effect size of the statistical learning effect (i.e., the RT difference for high- vs. low-probability location distractors) in the extinction phase was d = .47. We calculated the sample size using MorePower 6.0.4 (Campbell & Thompson, 2012), an alpha of 0.01 and power of 0.95. We found the minimum sample size to be 18 participants. However, our study required a larger sample size because its objective was not only to detect whether the color suppression effect survives in the extinction phase, but also to determine (a) whether the effect during learning will correlate with probability estimates and (b) whether the effect during extinction will be reduced in the informed relative to the uninformed group. To address the former question, we relied on Gimenez-Fernandez et al. (2020, Exp.2) who tested statistical learning of target location with 161 participants and found a significant correlation between the statistical learning effect during the learning phase and the % assigned by participants to the high-probability target quadrant. Accordingly, we tested 160 participants (80 in each group, informed and uninformed). With regard to the latter question, we had no previous literature to rely on. However, given that the sample required to detect the color suppression effect during extinction was 18 participants, we reasoned that more than four times as many participants (80 participants per group) should suffice to detect the interaction of this effect with group (informed vs. uninformed).

### Participants

One hundred and sixty participants (average age 27.3 years (SD = 4.6), 13 left-handed and 8 ambidextrous, 111 females), were recruited through the Prolific platform. Participation was limited to participants aged between 18 and 35 years, with English as their mother tongue, either normal or corrected-to-normal vision and no diagnosed attention disorders.

### Apparatus

The experiment was programmed on PsychoPy3 (Peirce et al., 2019) using the PsychoJS library and was hosted by Pavlovia (pavlovia.org). The experiment was allowed to run only on desktops or on laptops and exited automatically if a smartphone or tablet was detected. Participants were instructed not to exit full screen, but in case this occurred, the experiment was programmed to re-size itself to match the new window size. Responses were collected from the computer keyboard. The consistency of stimulus sizes across different display setups (screen resolution, screen size and viewing distances) was achieved using Li et al.’s (2020) credit card adjustment procedure: participants were asked to place their credit cards against a rectangle shown on the screen and to use the up and down arrow keys to resize the rectangle until it matched the size of the card.

### Stimuli

The search display contained a gray fixation cross in the center of the screen, surrounded by six shapes, three in each hemifield, placed equidistantly on an imaginary circle (2.6°) centered at fixation (see Figure 1). The target was the unique shape among homogenously shaped non-targets, either the unique circle (1.4° in diameter) among diamonds (1.2° × 1.2°) or vice-versa. Each shape was centered 2.6° from fixation and contained a gray line (either vertical or horizontal, 0.4° in length and 0.09° in width) in its center. On distractor-absent trials, all items were green. On distractor-present trials, one of the non-targets (the color singleton), had a different color, randomly chosen from five possible colors (yellow: RGB (255,255,0), red: (255,0,0), pink: RGB (255,192,203), blue: RGB (0,0,255), and orange: RGB (255,165,0). All stimuli were presented against a black background.

During the awareness test, an instruction screen read: “On what % of the trials do you think that the unique-color shape, when present, took on each of the following colors? Please type your estimate under each colored shape. Below these instructions, five outline shapes similar to those used during the search task appeared, each in a different possible color-singleton color, and arranged in one row. The order of the colors within the row varied randomly across participants. The shapes were either all diamonds or all circles, counterbalanced across participants. A window appeared under each shape, in which participants were able to type their answers.

### Procedure

*Search task*. Each search trial began with the fixation cross for 500 ms and was followed by the search display until response or for a maximum of 2,000 ms. A new trial began after a 500-ms blank inter-trial interval. Participants were asked to report the orientation of the gray line inside the target by pressing the “M” key when this line was horizontal and the “K” key when it was vertical, on the keyboard, as fast and as accurately as possible. An incorrect response was followed by a beep (225 Hz). Eye movements were not monitored but participants were explicitly instructed to maintain fixation. Participants were allowed a short self-pace break of a maximum of three minutes after each block of trials.

*Awareness test*. The instruction screen appeared after the last search trial, and participants were asked to estimate the percentage of times that the color distractor appeared in each of the five possible colors by typing a number between 0 and 100 under each color shape. They were instructed that the sum of the percentages assigned to the five colors should equal 100.

### Design

The search task was administered in three phases: a practice phase, followed by a learning phase and then by an extinction phase. The practice phase (first 20 trials) included only distractor-absent trials. In the learning phase (360 trials, divided into three epochs, each including two blocks of 60 trials each), the color singleton, when present (on 2/3 of the trials) appeared in one of the five colors (high-probability color) on 65% of the trials and in each of the other four colors (low-probability colors) on 8.75% of the trials. Which color served as the high-probability color remained the same for each participant throughout the learning phase and was counterbalanced across participants. In the extinction phase (360 trials, divided into three epochs, each including two blocks of 60 trials each), the color singleton appeared in each of the five colors with the same probability. In both phases, the target and distractor locations were randomly assigned on each trial but never overlapped. The line inside each shape was randomly either horizontal or vertical, with the constraint that in each display, exactly three lines were horizontal and three vertical. Conditions of distractor presence and distractor color were randomly mixed within each block of trials.

Participants were randomly assigned to two groups. The *informed* group underwent the awareness test immediately after the learning phase. Following the test, participants in this group were informed of the probability manipulation in the learning phase and of the fact that this manipulation would be discontinued in the next phase. The *uninformed* group underwent the awareness test after the extinction phase and was not informed of the transition between the two phases. The uninformed group included 78 participants and the informed group included 82.^4^

## Results

Any participant who responded on fewer than 90% of the trials or performed with less than 70% accuracy was automatically replaced with a new participant (21 participants). This exclusion procedure was not pre-registered but was implemented automatically due to default settings in our lab for RT-based online experiments. Error trials (12.5% and 9% of the trials in the learning and extinction phases, respectively) as well as RT outliers, defined as any correct-response trial with an RT differing from the mean of its cell by more than 2.5 standard deviations (1.7% and 2.2% of the remaining trials in the learning and extinction phases, respectively) were excluded from all RT analyses. Despite our instructions, the sum of the percentages assigned to the five colors by some of the participants did not equal 100. Their estimates were therefore converted to make the sum equal to 100 (henceforth, *converted estimates*). All RT analyses were conducted on the raw RTs but all the findings were replicated when the log-transformed RTs were used instead. Analyses that were not pre-registered are reported as exploratory.

### Learning phase

**Figure 2:**
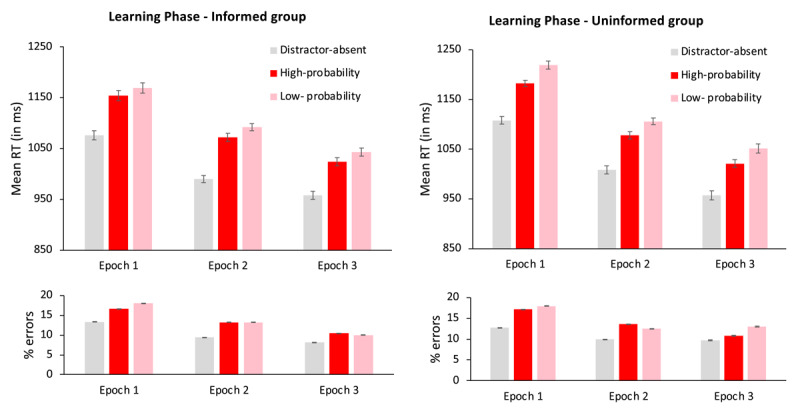
Mean reaction times (in milliseconds) in the learning phase, as a function of distractor condition (absent, present in the high-probability color and present in a low-probability color) and epoch (1–3), for the informed (left panel) and the uninformed (right panel) groups. Error bars denote within-subject standard errors (Morey, 2008).

We conducted an Analysis of Variance (ANOVA) on the data from the learning phase with distractor condition (absent, present in the high-probability color, or present in a low-probability color) and epoch (1 to 3) as within-subject variables and group (informed vs. uninformed) as a between-subject variable, separately for mean RTs and accuracy rates. Mean RTs and accuracy for the low-probability colors were calculated as the average across all four low-probability colors. The mean RT and error rate for each cell are presented in Figure 2.

*Reaction times*. The main effect of distractor condition was significant, F(2, 316) = 293, p < .0001, \eta _p^2 = .65. Planned comparisons showed that search was faster when the singleton distractor was absent than when it was present in the high-probability color, t(159) = 17.3, p < .0001, d = 1.37 or in a low-probability color, t(159) = 19.5, p < .0001, d = 1.54, indicating that the color-singleton distractor interfered with search performance in both the high-and the low-probability color conditions. Crucially, there was a significant color suppression effect – search was faster when the singleton distractor appeared in the high- than in a low-probability color, 23ms, t(159) = 6.2, p < .0001, d = .49, BF_10_ > 100.

The main effect of epoch was also significant, F(2, 316) = 217.4, p < .0001, \eta _p^2 = .58, with faster RTs as the experiment progressed. Epoch did not interact with distractor condition, F < 1. We conducted a finer-grained (pre-registered) analysis of the first epoch by dividing it into three parts (of 40 trials each).^5^ Table 1 shows the mean RTs. The interaction between color suppression and first epoch’s part was not significant, F < 1. Paired comparisons confirmed that color suppression was significant for the first, the second and the third parts of the first epoch, t(157) = 2.3, p = .02, d = .18, t(158) = 3.1, p = .002, d = .25, and t(159) = 2.4, p = .02, d = .19, respectively.

**Table 1:** Mean reaction times (RTs, in milliseconds) in the three parts (1–40 trials, 41–80 and 81–120 trials) of the first epoch of the learning phase, as a function of distractor condition (distractor absent, present in the high-probability color, and present in a low-probability color) and epoch (1–3). The numbers in brackets denote within-subject standard errors (Morey, 2008).


	TRIALS 1–40	TRIALS 41–80	TRIALS 81–120

Distractor-absent	1146 [9]	1083 [8]	1077 [8]

High-probability	1232 [9]	1142 [8]	1132 [9]

Low- probability	1258 [11]	1174 [10]	1155 [9]


The interaction between epoch and group was significant, F(2, 316) = 3.5, p = .03, \eta _p^2 = .02. Since participants were randomly assigned to the groups and underwent the exact same procedure during the learning phase, and since the effect size is negligible, we do not consider this finding any further. The interaction between distractor condition and group was not significant, and neither was the 3-way interaction with epoch, F(2, 316) = 1.2, p = .29, \eta _p^2 = .008 and F < 1, respectively.

*Accuracy*. The accuracy data mirrored the RT data, indicating that there was no speed-accuracy trade-off. The main effect of distractor condition was significant, F(2, 316) = 50.4, p < .0001, \eta _p^2 = .24. Planned comparisons showed that participants made fewer errors when the singleton distractor was absent vs. present in the high-probability color, t(159) = 8.9, p < .0001, d = .7 or in a low-probability color, t(159) = 8.6, p < .0001, d = .68. Color suppression was not significant, t < 1. The main effect of epoch was significant, F(2, 316) = 59, p < .0001, \eta _p^2 = .27, with fewer mistakes as the experiment progressed. This effect interacted with distractor condition, F(4, 632) = 3.24, p = .012, \eta _p^2 = .02. There was no color suppression effect in any of the epochs, all ps > .12, but the difference between distractor-absent and distractor-present conditions (across high- and low-probability color trials), though significant in all epochs, decreased in the third epoch relative to the second epoch, F(1,159) = 5, p = .027, \eta _p^2 = .03, with no difference between the first and second epochs, F < 1. These findings indicate that although there was no color suppression effect on accuracy in the learning phase, overall distractor interference decreased during the last epoch. The interaction between distractor condition and group and the three-way interaction between distractor condition, group and epoch were not significant, F < 1 and F(4, 632) = 1.5, p = .21, \eta _p^2 = .009.

#### Exploratory analysis: Inter-trial priming

We assessed the effect of distractor-color repetition (repeated vs. changed color relative to trial *n–1*) in a one-tailed t-test on trials in which the distractor was present on both the current and the previous trials. Unlike in the extinction phase where all distractor colors were equiprobable, it was not appropriate to conduct the analysis across conditions of distractor-color probability, because intertrial priming would be contaminated by the color probability effect (since color repetitions were more likely for the high- than for the low-probability colors). Therefore, only high-probability color trials were included in this analysis. There were not enough trials to conduct the same analysis on low-probability color trials.

*Reaction times*. Performance tended to be faster when the distractor color repeated than when it changed on successive trials, 1081 ms vs. 1089 ms, t(159) = 1.8, p = .08, d = .14.

*Accuracy*. The distractor-color repetition effect was not significant, t < 1.

### Extinction phase

**Figure 3:**
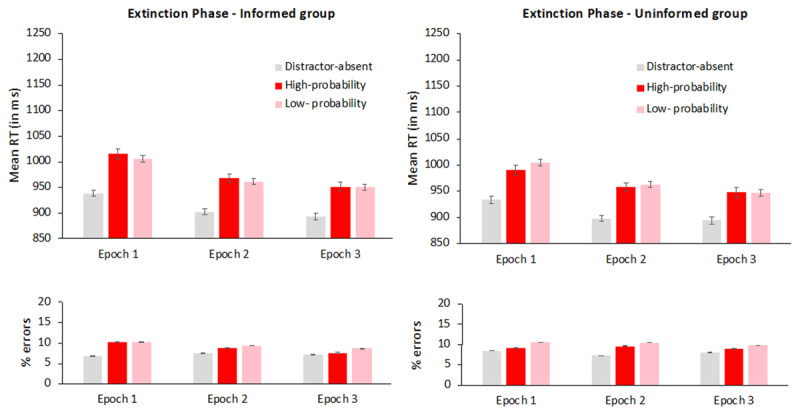
Mean reaction times (in milliseconds) in the extinction phase, as a function of distractor condition (absent, present in the high-probability color and present in a low-probability color) and epoch (1–3), for the informed (left panel) and the uninformed (right panel) groups. Error bars denote within-subject standard errors (Morey, 2008).

We conducted an ANOVA on the data from the extinction phase with distractor condition (absent, present in the high-probability color, or present in a low-probability color) and epoch (1 to 3) as within-subject variables and group (informed vs. uninformed) as a between-subject variable, separately for mean RTs and accuracy rates. The mean RT and error rate for each cell are presented in Figure 3.

*Reaction times*. The main effect of epoch was significant, F(2, 316) = 40, p < .0001, \eta _p^2 = .2, indicating that participants continued to become faster as the experiment progressed. The main effect of distractor condition was also significant, F(2, 316) = 168, p < .0001, \eta _p^2 = .52. Planned comparisons showed that search was faster when the singleton distractor was absent vs. present in the high-probability color, t(159) = 14.3, p < .0001, d = 1.1 or in a low-probability color, t(159) = 18.6, p < .0001, d = 1.5. Crucially, however, there was no significant color suppression, t < 1, BF_01_ = 17. In addition, distractor condition did not interact with group, F(2, 316) = 1.4, p = .24, \eta _p^2 = .009. There were no other significant effects, all Fs < 1.

*Exploratory analysis*: Although the 3-way interaction between group, epoch and color suppression was not significant, visual inspection of the numerical trends (see Figure 2) suggests that the color suppression effect was larger for the uninformed than for the informed group in the first epoch of the extinction phase (15 ms vs. –10 ms, respectively), a difference that gradually disappeared in the second (5 ms vs. –7 ms), and third (1 ms vs. 1 ms) epochs. To examine whether this observation was statistically reliable, we assessed the interaction between color suppression and group, separately for each epoch. This interaction indeed approached significance during the first epoch, F(1, 158) = 3.3, p = .07, \eta _p^2 = .02 and became non-significant for the second and third epochs, both Fs < 1. To examine whether the two groups might differ more reliably at the very beginning of the first epoch, we performed a finer-grained analysis of the first epoch by dividing it into three parts (of 40 trials each). The interaction between color suppression and group was not significant for any of the first epoch’s parts, all ps > .18.

*Accuracy*. The main effect of distractor condition was significant, F(2, 316) = 24.4, p < .0001, \eta _p^2 = .52. Planned comparisons showed that participants made fewer mistakes when the singleton distractor was absent vs. present in the high-probability color, t(159) = 3.7, p = .0002, d = .29 and in a low-probability color, t(159) = 8.1, p < .0001, d = .64. Crucially, there was a small but significant color suppression, t(159) = 3.01, p = 0.003, d = .24: participants committed fewer errors when the distractor was in the high- than in a low-probability color. Distractor condition did not interact with group, F < 1, and in particular, color suppression did not interact with group, F < 1. The main effect of epoch did not reach significance, F(2, 316) = 2.35, p = 0.1, \eta _p^2 = .2 but showed a numerical trend that mirrored the RT effect. There were no other significant effects. In particular, the three-way interaction between distractor condition, epoch and group was not significant, F(4, 632) = 1.72, p = .14, \eta _p^2 = .01, all other Fs < 1.

#### Inter-trial priming analysis

We assessed the effect of distractor-color repetition (repeated vs. changed relative to trial *n–1*) in a one-tailed t-test.^6^

*Reaction times*. Reaction times were faster when the distractor color repeated than when it changed on successive trials, 961ms vs. 969ms, t(159) = 2.1, p = .038, d = .17. A similar comparison for repetitions from trial *n–2* (excluding trials in which the distractor color repeated from trial *n–1*) did not yield a significant effect, t < 1.

*Accuracy*. The distractor-color repetition effect was not significant, t < 1.

### Awareness test

We examined the correlation between our measure of the participants’ awareness and the color suppression effect. To do that, we computed the Pearson correlation between the converted estimates of the percentage of high-probability distractor-color trials (collected after the learning phase for the informed group and after the extinction phase for the uninformed group) and the color-suppression effect during the last three blocks (i.e., the second half) of the learning phase. To increase power, this correlation was first tested across the two groups.

**Figure 4:**
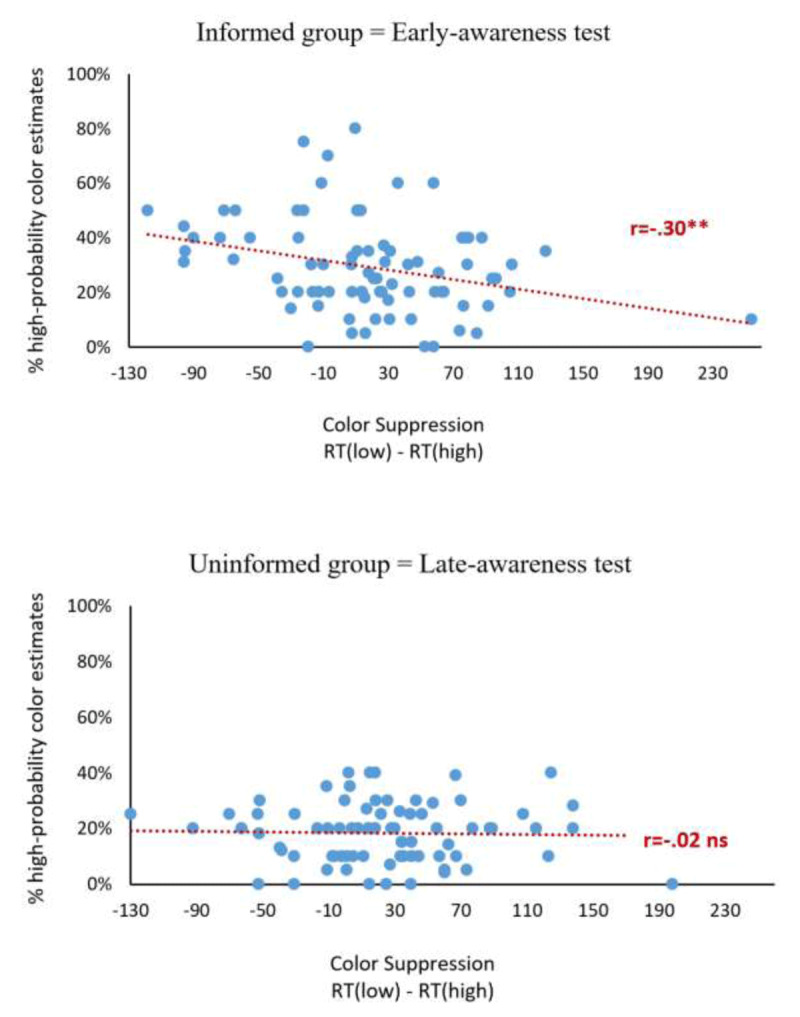
Scatter plot showing the correlation between the color suppression effect for the second half of the learning phase (x-axis) and the percentage assigned to the high-probability color (y-axis) for the informed group (upper panel) and the uninformed group (lower panel).

This analysis revealed that color suppression was higher, the *less aware* the participants were of the statistical contingency, r = –.21, t(158) = –2.71, p = .007. Next, we measured the correlation separately for the informed and uninformed groups, r = –.30, t(80) = –2.85, p = .006^7^ and r = –.03, t(76) = –0.27, p = .79, respectively (see Figure 4). Exploratory analyses on the normalized color suppression effects (RT(low)+RT(high)/RT(low)-RT(high)) closely replicated these findings.

Finally, we compared the mean converted proportion assigned to the high-probability color in the informed vs. non-informed group by conducting a one-tailed independent-samples *t* test. We found that participants who were tested immediately after learning (informed group) assigned higher ratings to the high-probability color than did participants who were tested after extinction (non-informed group), 29% vs. 18%, respectively, t(136) = 4.97, p < .0001, d = .78. Furthermore, while the estimates of participants in the informed group were above the 20% chance level, t(81) = 4.98, p < .0001, d = .77, the estimates of participants in the uninformed group did not differ from chance, t(77) = –1.43, p = .92, d = –.23.

## Discussion

Statistical learning of the most frequent property of a search target or of a salient distractor (such as their most probable location or color) is thought to be implicit, long-lasting, and inflexible (e.g., Jiang, 2018, Theeuwes, 2018). Here, we evaluated these claims with regard to statistical learning of distractor color. We found the learned bias to have a relatively small impact on observers’ ability to ignore the salient distractor, as it reduced interference from this distractor by about one third. Moreover, part of the effect resulted from the frequent intertrial repetitions of the high-probability distractor color. Crucially, we found the bias to be short-lived, a finding that limited the opportunity to determine whether it can be flexibly and purposefully withheld. Finally, our findings show that learned color suppression was implicit, as awareness of the probability manipulation reduced the bias rather than enhancing it.

### How robust and long-lasting is statistical learning of distractor color?

We used the additional singleton paradigm and manipulated the probability of the singleton distractor’s color following the procedure used by Stilwell et al. (2019, Exp. 2): the color singleton, when present could take on one of 5 colors and one of these colors was more than 7 times more likely than each of the remaining colors. We replicated Stillwell et al.’s color suppression finding with a much larger sample (160 here vs. 24 participants in Stilwell et al., 2019, Exp.2): across the learning phase, participants were faster when the singleton distractor appeared in the high- than in a low-probability color. However, this color suppression effect (23 ms, Cohen’s d = .49) was smaller than in the original study (39 ms)^8^ – although distractor interference was of similar magnitude in the two studies.

The color suppression effect directly attributable to statistical learning in the learning phase was actually even smaller: part of it was accounted for by inter-trial priming of the distractor’s color, which was significant in the extinction phase, albeit small (d = .14). We had argued that excluding repetitions from the previous trial to control for contamination of statistical learning by intertrial priming during the learning phase may not be enough, based on previous findings showing that trials from 5 to 8 trials back have a substantial influence on search performance (e.g., Maljkovic & Nakayama, 1994; 1996 for intertrial priming of target color and target location). Here, we found no evidence for intertrial priming beyond the previous trial. However, this null effect is likely due to the fact that intertrial priming from the previous trial was already rather small in our study. Thus, for statistical learning of other properties for which intertrial priming is larger (e.g., statistical learning of target feature or location), the contribution of intertrial priming may nevertheless be strongly underestimated if only repetitions from the previous trial are taken into account.

Interestingly, the color-suppression bias was already present in the first epoch and did not increase further across the next two epochs. A finer-grained analysis of the first epoch of the learning phase showed that the bias was absent across the first 40 trials but already reached its maximum over the next 40 trials. Thus, at least with the strong probability imbalance implemented here (65% vs. 8.75% for the high- vs. low-probability color, respectively), the bias developed very fast. No previous study examined the time course of statistical learning of distractor color, but for comparison, statistical learning of distractor location with a similar probability imbalance (40% and 6.6% for the high- vs. low-probability locations, respectively) was found to occur similarly fast, within the first 75 trials (Valsecchi & Turatto, 2021).

Crucially, we found little evidence for the notion that color suppression survived during extinction. The effect on accuracy was highly significant but did not exceed 1% (d = .24).

Taken together, these results suggest that unlike statistical learning of distractor location (e.g., Britton & Anderson, 2020; Duncan & Theeuwes, 2020), statistical learning of distractor color has a rather small and short-lived effect on search performance.

These findings are surprising in light of the vast literature showing that when a color characterizes a distractor on 100% of the trials on which this distractor is present, suppression is very robust (see Gaspelin & Luck, 2019; Geng et al., 2019 for recent reviews on distractor inhibition). For instance, Vatterott and Vecera (2012) had observers search for a shape target and a color-singleton distractor, the color of which changed at the beginning of each block of trials, was present on 50% of the trials. The results showed that while the presence of the color singleton strongly interfered with search during the first half of each block, it actually improved performance on the second half of each block. The authors concluded that experience with a specific color singleton distractor allows observers to effectively suppress this distractor’s color. In addition, Vecera et al. (2014) reported that a distractor color that observers learned to ignore remained suppressed even after the observers learned to suppress other salient-color distractors, during intervening blocks of trials – a finding suggesting that color-based suppression is long-lasting. Why was experience-based distractor rejection so much less dramatic in the present study than in Vatterott and Vecera’s (2012) and other distractor-inhibition studies?

A possible explanation for this difference is that in our study, which was modeled after Stilwell et al.’s (2019), participants searched for a known shape singleton, whereas in distractor-inhibition studies, participants typically look for a shape target among heterogeneously shaped non-targets. As a result, in our study participants could use singleton-detection mode (i.e., the strategy of searching for an object with a unique feature, Bacon & Egeth, 1994), whereas in distractor-inhibition studies such as Vatterott and Vecera (2012), they had to use feature-search mode. Therefore, statistical learning of distractor color (e.g., Stilwell et al., 2019) and color-based distractor inhibition (e.g., Vatterott et al., 2018) may index the same experience-based distractor rejection mechanism, but such mechanism may be less effective in singleton search than in feature search. Note in this regard that our findings, as well as Stilwell et al.’s (2019), are inconsistent with recent findings by Tommaso and Turatto (2019) suggesting that experience-based distractor inhibition does not occur in singleton search. Further research is needed to resolve this discrepancy.

### Is statistical learning of a salient distractor’s color implicit?

Our study yielded important insights into the role of awareness of the probability manipulation in statistical learning. The first insight is methodological: we showed that testing awareness of the statistical imbalance after the extinction phase (e.g., Jiang et al., 2013, Ferrante et al., 2018) strongly underestimates such awareness, in line with Gimenez-Fernandez et al.’s (2020) claim, and yields noisy data: the early awareness-test group (who were asked to report their distractor-color percentage estimates after the learning phase) provided significantly higher estimates for the high-probability color than the late awareness-test group (who were tested after extinction), 29% vs. 18%, respectively. Notably, the latter group did not perform better than chance, a finding typically held to indicate that the observers are unaware of the probability manipulation (e.g., Jiang et al., 2013) – whereas the former group was above chance. In addition, while probability estimates were significantly correlated with color suppression during the learning phase for the early-awareness group, such correlation was entirely absent for the late-awareness group.

Crucially, however, the finding that in our study participants had some awareness of the high-probability color does not imply that statistical learning was contingent on such awareness. Indeed, we found the correlation between probability estimates and color suppression to be *negative*: consciously noticing that one color distractor was more frequent than the others made it difficult for observers to ignore it. This observation extends previous findings showing that explicitly informing participants to ignore a distractor leads to more rather than to less distraction by this distractor, a phenomenon known as the “cued distraction” or “attentional white-bear” effect (Moher & Egeth, 2012; Stilwell & Vecera, 2019a; 2019b, see also Tsal & Makovski, 2006; Wegner, Schneider, Carter, & White, 1987). Here, we showed that this effect can occur in the absence of explicit instructions. Specifically, we showed that simply noticing that a to-be-ignored salient distractor was more likely to appear in a particular color led participants to pay more attention to distractors that matched that high-probability color than to distractors with a different low-probability color.

From a theoretical viewpoint, the main implication of this finding for the present purposes in that statistical learning can be implicit, since awareness of the probability manipulation and the effects of this manipulation (color suppression) were dissociated in our study: awareness of the high-probability color increased the priority of that color, whereas statistical learning reduced it. Learned color suppression cannot, therefore, be contingent on awareness of the probability manipulation. Note that such dissociation cannot be uncovered in studies of *target* statistical learning because, by design, the probability manipulation and awareness of that manipulation influence performance in the same direction: they both increase the target’s priority (e.g., Gimenez-Fernandez et al., 2020).

From a methodological viewpoint, our findings illustrate the advantage of using the continuous measure of awareness pioneered by Vadillo and colleagues (Vadillo et al., 2020; Gimenez-Fernandez et al., 2020) and highlight the benefits of complementing this measure with correlational approach in the study of distractor statistical learning (Vadillo et al., 2020) – in a novel way: this approach allowed us to demonstrate that even though participants had some awareness of the manipulation (indicating that this manipulation was not subtle enough to go fully unnoticed), learning was implicit. Finally, in line with Gimenez-Fernandez et al.’s (2020) work, our findings strongly indicate that post-extinction awareness tests should be abandoned in future research on statistical learning.

### Is statistical learning of a salient distractor’s color inflexible?

Our last question was about the flexibility of distractor-color statistical learning. We asked whether informing participants that the probability manipulation would be discontinued during the upcoming phase would reduce the bias during extinction. However, the bias was virtually absent in the extinction phase, and it was therefore difficult to detect any possible difference between the informed and uninformed group: a difference in the expected direction (i.e., less color suppression in the informed vs. uninformed group) emerged only during the first epoch and only approached significance. To test the flexibility of statistical learning of distractor color, further research is needed, with a stronger manipulation of color suppression, for instance, during feature search instead of singleton search.

## Conclusions

In line with previous research, we found that observers can learn to ignore a salient distractor that appears most frequently in a given color. We showed for the first time that such bias is implicit. Although part but not all of the bias away from the high-probability color could be explained by repetition effects unrelated to learning, we found the learnt color suppression to be short-lived. This finding stands in contrast with ample research showing that learned suppression of high-probability distractor *locations* survives after the probability manipulation is discontinued (e.g., Britton & Anderson, 2020; Duncan & Theeuwes, 2020; Valsecchi & Turatto, 2021). Further research is needed to determine whether there are qualitative differences between feature-based and location-based regularities in the control of attention.

## Data Accessibility Statements

This study’s design and its analyses were pre-registered. All data, analysis scripts, and stimulus materials are posted on the Open Science Framework repository and can be accessed at https://osf.io/rs3z9/.

## Footnotes

Here, we focused on the studies in which the distractor was salient (but see Lien, Ruthruff and Hauck (2022) for evidence suggesting that salience may not be necessary for suppression to occur).

In the same study, they showed that when participants were asked to rank each quadrant of the visual display (from 1 to 4) according to how often they thought the target had appeared there (Exp. 1) the statistical learning effect did not correlate with the rankings.

It is more appropriate to investigate the role of inter-trial priming during the extinction phase than during the learning phase, because the probability of a distractor-color repetition is the same for the (previously) high- and low-probability colors only in the former. Accordingly, the analysis of distractor color intertrial priming was pre-registered only for the extinction phase. Nevertheless, we conducted a similar analysis on the data of the learning phase, as an exploratory investigation.

We discovered this slight imbalance after the data collection was terminated.

Two participants were excluded from these analyses because they had no data in one of the analysis cells.

We had planned to conduct an ANOVA with distractor-color repetition (repeated vs. changed relative to trial *n–i*) and distractor-color probability on trial *n* (high- vs. low-probability color) as within-subject variables and group (informed vs. uninformed) as a between-subjects variable, but we realized that there were not enough trials in each cell to conduct a meaningful analysis. We therefore performed only a paired comparison between repeated vs. changed distractor color in order to determine whether any intertrial priming of distractor color could be observed.

Visual inspection of the scatter plot suggests that there was an outlier participant in the informed group. After removing this participant, the correlation remained significant, r = –.28, t(79) = –.2.6, p = .01.

Since the original study did not report the effect size, we can only refer to the difference in overall RTs between the low- and high-probability color trials).
